# Risk prediction model for non-exclusive breastfeeding within 6 weeks postpartum in primiparous women

**DOI:** 10.3389/fmed.2026.1842493

**Published:** 2026-05-07

**Authors:** Lina Yang, Xiaohong Zhu, Wenjie Wang, Chunling Bao, Rui Fang, Longfang Ni, Rui Tai, Mengru Liu, Cuiqin Huang, Rong Huang

**Affiliations:** 1 Department of Nursing, Shanghai Sixth People’s Hospital Affiliated to Shanghai Jiao Tong University School of Medicine, Shanghai, China; 2 Department of Nursing, Shanghai General Hospital, Shanghai Jiao Tong University School of Medicine, Shanghai, China; 3 Department of Obstetrics and Gynecology, Shanghai Sixth People's Hospital Affiliated to Shanghai Jiao Tong University School of Medicine, Shanghai, China; 4 School of Nursing, Bengbu Medical College, Bengbu, China; 5 Department of Nursing, Shanghai Key Laboratory of Maternal Fetal Medicine, Shanghai Institute of Maternal-Fetal Medicine and Gynecologic Oncology, Shanghai First Maternity and Infant Hospital, School of Medicine, Tongji University, Shanghai, China

**Keywords:** breastfeeding support, non-exclusive breastfeeding, postpartum period, primiparous women, risk prediction model

## Abstract

**Objective:**

To examine the occurrence of non-exclusive breastfeeding within 6 weeks postpartum in primiparous women and to establish a risk prediction model for early identification of women at high risk.

**Methods:**

In this single-center prospective cohort study, primiparous women who delivered at a tertiary general hospital in Shanghai, China, between July 2024 and July 2025 were consecutively recruited by convenience sampling. Baseline information was collected during postpartum hospitalization, and infant feeding patterns were followed weekly for 6 weeks after discharge. Candidate predictors were derived from self-designed questionnaires and standardized scales. Univariable analyses and multivariable logistic regression were performed to identify factors associated with non-exclusive breastfeeding within 6 weeks postpartum. Model performance was assessed by the likelihood ratio test, Hosmer–Lemeshow goodness-of-fit test, and receiver operating characteristic analysis.

**Results:**

Among the 650 primiparous women included in the study, 322 developed non-exclusive breastfeeding within 6 weeks postpartum, corresponding to an incidence of 49.53%. Immediate postpartum latching, amniotic fluid abnormality, Social and Professional Support, Positive Breastfeeding Sentiment, total breastfeeding behavior score, total infant feeding intention score, and total breastfeeding knowledge score were independently associated with non-exclusive breastfeeding within 6 weeks postpartum (all *p* < 0.05). The model demonstrated good fit (likelihood ratio χ^2^ = 258.368, *p* < 0.001; Hosmer–Lemeshow χ^2^ = 10.601, *p* = 0.330) and good discrimination (AUC = 0.841, 95% confidence interval: 0.811–0.871).

**Conclusion:**

Non-exclusive breastfeeding within 6 weeks postpartum was common in primiparous women. The proposed prediction model showed good performance and may provide a useful tool for early risk stratification and targeted breastfeeding support in clinical practice.

## 1 Introduction

Breastfeeding is a cornerstone of maternal and infant health, and the exclusive breastfeeding rate is also an important indicator of the quality of maternal and child health services (1). Previous studies have shown that maintaining exclusive breastfeeding for the first 6 months of life provides the greatest health benefits for both mothers and infants (2). The World Health Organization (WHO) and the United Nations Children’s Fund (UNICEF) also recommend initiating breastfeeding within 1 h after birth and maintaining exclusive breastfeeding during the first 6 months of life (3).

In practice, however, early discontinuation of exclusive breastfeeding and premature introduction of supplementary feeding are common. Buttham et al. reported that breastfeeding difficulties occur predominantly in the early postpartum period, and that the decline in exclusive breastfeeding is most pronounced during the first few weeks after birth. A substantial proportion of women who do not ultimately maintain exclusive breastfeeding have already changed their feeding pattern within 6 weeks postpartum (4). Therefore, 6 weeks postpartum represents a meaningful time point for evaluating early breastfeeding outcomes. Assessing breastfeeding status at this stage may help identify women at high risk of non-exclusive breastfeeding early in the period when exclusive breastfeeding declines most markedly, and may also provide a basis for earlier clinical intervention.

In early postpartum breastfeeding support, the risk of non-exclusive breastfeeding is not the same across women. Primiparous women, because they lack previous breastfeeding experience, are more likely to be affected during breastfeeding establishment by multiple factors, including individual cognition, maternal and infant conditions, family support, and healthcare support. They therefore represent a key target population in breastfeeding management (5). Existing studies have mainly focused on individual factors associated with breastfeeding or on prediction models developed for other postpartum time points (6, 7). However, breastfeeding is a dynamic and evolving process, and the relevant factors and their predictive effects may vary across stages (8). Specifically, three key gaps in existing research remain to be addressed. First, most existing studies on breastfeeding risk prediction focus on 3 months, 6 months or longer postpartum endpoints (9, 10), with very few studies specifically targeting the 6-week postpartum window, which has been confirmed as the period with the most significant decline in exclusive breastfeeding (11). Second, existing studies mostly focus on the independent association between single factors and breastfeeding outcomes (12), while few studies have integrated perinatal clinical indicators, social support, breastfeeding attitude, feeding behavior, feeding intention and breastfeeding knowledge into the analysis of breastfeeding risk in the early postpartum period (13). Third, for primiparous women, the key population for breastfeeding management, studies specifically focusing on the prediction of non-exclusive breastfeeding within 6 weeks postpartum are still very limited (14), and there is a lack of corresponding risk prediction models, which makes it difficult to timely identify high-risk women and implement stratified management and targeted support in clinical practice.

Accordingly, this study focused on primiparous women to investigate the occurrence of non-exclusive breastfeeding within 6 weeks postpartum, identify the independent factors associated with this outcome, and develop a risk prediction model. The aim was to support early risk stratification, discharge education, and follow-up planning in routine postpartum nursing care.

## 2 Methods

### 2.1 Study design

This was a single-center prospective cohort study to identify factors associated with non-exclusive breastfeeding within 6 weeks postpartum among primiparous women and to develop a risk prediction model. The study was designed and reported in accordance with the Strengthening the Reporting of Observational Studies in Epidemiology (STROBE) Statement (15).

### 2.2 Study setting and participants

Using convenience sampling, we consecutively recruited primiparous women who delivered at the Department of Obstetrics and Gynecology of a tertiary general hospital in Shanghai, China, between July 2024 and July 2025. Baseline data were collected during postpartum hospitalization, and all participants were followed up for 6 weeks after discharge. The inclusion criteria were as follows: (1) age ≥20 years and antenatal care records established at the study hospital; (2) primiparous women with a singleton pregnancy; (3) ability to communicate normally, understand the questionnaire content, and complete the survey; (4) willingness to participate and provision of written informed consent; and (5) availability for follow-up via telephone or WeChat. The exclusion criteria were as follows: (1) use of medications during pregnancy or postpartum that might affect lactation; (2) a history of psychiatric disorders, intellectual disability, or severe psychological abnormalities; (3) neonates with sucking dysfunction or confirmed contraindications to breastfeeding; and (4) severe obstetric complications that precluded breastfeeding.

### 2.3 Sample size estimation

The sample size was preliminarily estimated using the events per variable (EPV) method (16). Based on the literature review and preliminary work, approximately 27 candidate predictors were prespecified. According to the rule that at least 15 outcome events are required for each candidate predictor in a logistic regression model, at least 405 outcome events were needed during the model development stage. Given that the incidence of non-exclusive breastfeeding within 6 weeks postpartum was approximately 72% in the pilot survey, the minimum analyzable sample size was estimated to be 563. After accounting for a 10% loss to follow-up rate, at least 620 participants were required.

### 2.4 Predictors and measurement

Candidate predictors were collected during postpartum hospitalization using a self-designed questionnaire for general and clinical information together with standardized scales. The candidate predictors were grouped into the following three categories.

1. Sociodemographic and family support-related variables

Age, educational level, marital status, occupation, monthly household income, method of medical expense payment, breastfeeding plan, ability to continue breastfeeding after returning to work, primary postpartum caregiver, whether the feeding plan had been discussed with the spouse, attention to infant formula information, number of anticipated reasons for discontinuing breastfeeding, and number of breastfeeding knowledge sources were collected from participant-reported information. Age was treated as a continuous variable in years, whereas the remaining variables were recorded according to predefined categories. The variable “attention to infant formula information” was defined as whether the participant had paid attention to or actively sought infant formula-related information during pregnancy or postpartum hospitalization. This variable was recorded as a dichotomous yes/no item and was intended to reflect attention to or exposure to such information only, rather than knowledge about formula feeding, agreement with formula use, maternal beliefs, or receipt of free samples.

1. Perinatal maternal and infant clinical variables

Mode of delivery, gestational age, neonatal birth weight, neonatal sex, 1-min Apgar score, duration of mother–infant skin-to-skin contact after birth, immediate postpartum latching, onset of lactogenesis II, history of breast surgery, umbilical cord abnormalities, amniotic fluid abnormalities, number of prepregnancy comorbidities, and number of pregnancy complications were collected. These variables were prespecified as candidate perinatal clinical predictors because they are routinely documented obstetric indicators and may reflect fetal well-being or intrapartum conditions relevant to early breastfeeding establishment. In particular, umbilical cord abnormalities and amniotic fluid abnormalities have been reported to be associated with adverse obstetric or perinatal outcomes, while pregnancy-related complications may delay early initiation of breastfeeding; moreover, meconium-stained amniotic fluid has been linked to feeding problems at the first feed. Therefore, these variables were included in the univariable analysis as clinically plausible candidate predictors rather than on the assumption that they were already established independent predictors of non-exclusive breastfeeding within 6 weeks postpartum. These data were mainly obtained from medical records and maternal questionnaires (6, 7). Immediate postpartum latching was defined as achievement of effective latching within 1 h after birth and was recorded as yes or no. Timely onset of lactogenesis II was defined as the appearance within 72 h postpartum of characteristic signs such as a marked increase in milk production and breast fullness and was recorded as timely onset or delayed onset. History of breast surgery referred to any previous breast-related surgery and was recorded as yes or no. Umbilical cord abnormalities and amniotic fluid abnormalities were recorded as yes or no according to obstetric records. The numbers of prepregnancy comorbidities and pregnancy complications were recorded according to the actual number of diagnosed conditions.

1. Scale score variables

These included the Breastfeeding Attrition Prediction Tool (BAPT) (17), the Breastfeeding Knowledge Questionnaire (BKQ) (18), and the Infant Feeding Intentions Scale (IFI) (19).

The BAPT was a revised Chinese version comprising 48 items across four dimensions and scored on a 5-point Likert scale: Social and Professional Support (SPS; 11 items), Negative Breastfeeding Sentiment (NBS; 15 items), Breastfeeding Control (BFC; 10 items), and Positive Breastfeeding Sentiment (PBS; 12 items). The overall Cronbach’s *α* coefficient was 0.903. Scores for each dimension were entered separately into the analysis as continuous variables.

The BKQ consists of 18 items in a true or false format. Each correct response is assigned 1 point and each incorrect response 0 points, yielding a total score ranging from 0 to 18. Higher scores indicate better breastfeeding knowledge. The Cronbach’s *α* coefficient of the questionnaire was 0.820.

The IFI consists of 5 items scored on a 5-point Likert scale, with a total score ranging from 0 to 16. Higher scores indicate stronger maternal intention to initiate and maintain exclusive breastfeeding. The overall Cronbach’s *α* coefficient was 0.90.

Breastfeeding behavior was assessed using the Breastfeeding Behavior Questionnaire developed by Alami Ali (20), a standardized questionnaire based on the Theory of Planned Behavior. The instrument includes breastfeeding intention (three items) and breastfeeding behavior (four items). In the present study, because infant feeding intention was assessed separately using the IFI, only the four breastfeeding behavior items were used to calculate the total breastfeeding behavior score, with higher scores indicating more positive breastfeeding behavior. The reported content validity range of the questionnaire is 0.65–0.99, Cronbach’s *α* coefficient is 0.79, and the intra-group correlation coefficient is 0.81.

The primary outcome was the occurrence of non-exclusive breastfeeding within 6 weeks postpartum. Non-exclusive breastfeeding was defined as failure to maintain exclusive breastfeeding continuously during the first 6 weeks postpartum.

### 2.5 Data collection and quality control

Before the formal investigation, a pilot survey involving 30 participants was conducted to assess the comprehensibility of the survey items, the time required to complete the questionnaire, and the feasibility of the overall survey procedure. The final questionnaire was revised and refined accordingly.

During the formal investigation, baseline data were collected face to face by trained investigators during postpartum hospitalization. Standardized instructions were used throughout the survey process. For participants who were unable to complete the questionnaire independently, investigators administered the questionnaire item by item and recorded the responses faithfully to ensure data completeness and consistency.

After discharge, participants were followed up weekly by telephone or WeChat from postpartum week 1 to week 6, and changes in infant feeding patterns were recorded dynamically. This short-interval, high-frequency follow-up strategy was adopted to shorten the recall window and minimize information bias associated with prolonged recall.

All data were independently entered by two trained investigators and cross-checked. For records with logical inconsistencies, obvious abnormalities, or substantial missing data, the original source documents were reviewed promptly and corrections were made where possible; invalid data were excluded when necessary to ensure the accuracy and completeness of the dataset.

### 2.6 Bias control

To reduce selection bias, all eligible participants within the prespecified study period were consecutively recruited, and uniform inclusion and exclusion criteria were applied throughout the study.

To minimize information bias and measurement bias, all investigators received standardized training before the formal survey. Standardized instructions, the same scale versions, and consistent interpretation criteria were used throughout data collection. In addition, double data entry and cross-checking were performed to reduce data entry errors.

To reduce recall bias, weekly follow-up was conducted during the first 6 weeks postpartum, thereby shortening the recall period for breastfeeding behaviors and reducing information distortion caused by long-term recall.

To reduce loss-to-follow-up bias, valid contact information was verified before follow-up, and participants were tracked through both telephone and WeChat. Those who did not respond on time were contacted again to maximize follow-up completion.

### 2.7 Statistical analysis and model construction

Participants were categorized into an exclusive breastfeeding group and a non-exclusive breastfeeding group according to whether non-exclusive breastfeeding occurred within the first 6 weeks postpartum. Non-exclusive breastfeeding was defined as failure to maintain exclusive breastfeeding continuously during this period.

All statistical analyses were performed using IBM SPSS Statistics version 26.0. Continuous variables were first tested for normality. Normally distributed continuous variables were expressed as mean ± standard deviation (SD), and between-group comparisons were performed using the independent-samples *t* test. Non-normally distributed continuous variables were expressed as median (interquartile range, IQR), and between-group comparisons were performed using the Mann–Whitney *U* test. Categorical variables were expressed as frequency and percentage, and between-group comparisons were performed using the chi-square test or Fisher’s exact test, as appropriate.

The occurrence of non-exclusive breastfeeding within the first 6 weeks postpartum was set as the dependent variable. Univariable analyses were first performed for all candidate variables, and variables with statistically significant differences (*p* < 0.05) were entered into a multivariable logistic regression model to identify the independent factors associated with non-exclusive breastfeeding among primiparous women within the first 6 weeks postpartum. Continuous variables were entered into the model using their original values; binary variables were coded as 0 and 1; and multicategory variables were transformed into dummy variables before analysis. Regression results were reported as the regression coefficient (*β*), standard error (SE), Wald chi-square value, odds ratio (OR), and 95% confidence interval (CI).

Overall model significance was assessed using the likelihood ratio test, and model calibration was evaluated using the Hosmer–Lemeshow goodness-of-fit test. In addition, receiver operating characteristic (ROC) curves were plotted based on the predicted probabilities generated by the logistic regression model, and the area under the curve (AUC) with its 95% CI was calculated to assess model discrimination. All statistical tests were two-sided, and *p* < 0.05 was considered statistically significant.

### 2.8 Ethics

This study was approved by the Ethics Committee of Shanghai Sixth People’s Hospital Affiliated to Shanghai Jiao Tong University School of Medicine (approval number: 2024-KY-074(K)). Written informed consent was obtained from all participants after they had been fully informed about the study. The principles of informed consent, voluntariness, confidentiality, and nonmaleficence were strictly observed throughout the study.

## 3 Results

### 3.1 Univariate analysis

A total of 650 primiparous women were ultimately included in this study, among whom 322 experienced non-exclusive breastfeeding within 6 weeks postpartum, yielding an incidence of 49.53%. Univariable analyses were performed for demographic characteristics, feeding-related behaviors, and feeding intentions. Immediate postpartum latching, history of breast surgery, amniotic fluid abnormality, timely onset of lactogenesis II, number of pregnancy complications, SPS, PBS, BFC, total breastfeeding behavior score, total infant feeding intention score, and total breastfeeding knowledge score showed statistically significant differences (all *p* < 0.05), as shown in Table 1.

**Table 1:** Univariable analysis of factors associated with the risk of non-exclusive breastfeeding within 6 weeks postpartum among primiparous women.

Item	Category	Exclusive Breastfeeding	Non-Exclusive Breastfeeding	Statistic *t*/χ^2^/Z	*P*
Education level [*n* (%)]	Junior High School and below	25 (7.6)	31 (9.6)	1.339	0.720
	High School/Secondary School	30 (9.1)	30 (9.3)		
	Associate/Bachelor’s Degree	215 (65.5)	199 (61.8)		
	Master’s Degree and above	58 (17.7)	62 (19.3)		
Marital status [*n* (%)]	Married	326 (99.4)	317 (98.4)	1.356	0.244
	Unmarried	2 (0.6)	5 (1.6)		
Household income (RMB/Yuan) [n (%)]	5,000–10,000	38 (11.6)	53 (16.5)	3.701	0.157
	10,001–20,000	156 (47.6)	141 (43.8)		
	>20,001	134 (40.9)	128 (39.8)		
Occupation [n (%)]	Worker/Employee	147 (44.8)	159 (49.4)	2.914	0.572
	Freelancer/Self-employed business owner	26 (7.9)	18 (5.6)		
	Administrative/Managerial staff	41 (12.5)	44 (13.7)		
	Unemployed/Job seeker	67 (20.4)	62 (19.3)		
	Professional/Technical personnel	47 (14.3)	39 (12.1)		
Payment method [*n* (%)]	Urban employee basic medical insurance	246 (75.0)	261 (81.1)	6.101	0.107
	New rural cooperative medical scheme	19 (5.8)	12 (3.7)		
	Urban resident basic medical insurance	24 (7.3)	12 (3.7)		
	Self-Pay	39 (11.9)	37 (11.5)		
Immediate postpartum latching [*n* (%)]	No	229 (69.8)	296 (91.9)	51.131	< 0.001
	Yes	99 (30.2)	26 (8.1)		
History of breast surgery [*n* (%)]	No	326 (99.4)	303 (94.1)	14.549	< 0.001
	Yes	2 (0.6)	19 (5.9)		
Umbilical cord abnormality [*n* (%)]	No	298 (90.8)	302 (93.8)	1.971	0.160
	Yes	30 (9.2)	20 (6.2)		
Amniotic fluid abnormality [*n* (%)]	No	257 (78.8)	273 (85.3)	4.461	0.035
	Yes	71 (21.2)	49 (14.7)		
Infant sex [*n* (%)]	Male	160 (48.8)	176 (55.0)	2.248	0.134
	Female	168 (51.2)	146 (45.0)		
Timely onset of lactogenesis II [*n* (%)]	Delayed onset	99 (30.2)	137 (42.8)	10.741	< 0.001
	Normal onset	229 (69.8)	185 (57.2)		
Breastfeeding plan [*n* (%)]	Mixed feeding	24 (7.3)	13 (4.0)	6.560	0.087
	Exclusive breastfeeding for <6 Months	9 (2.7)	4 (1.2)		
	Exclusive breastfeeding intended for 6 months	264 (80.5)	281 (87.3)		
	Never considered	31 (9.5)	24 (7.5)		
Ability to continue breastfeeding after returning to work [*n* (%)]	Yes	171 (52.1)	175 (54.3)	0.379	0.827
	No	94 (28.7)	86 (26.7)		
	Not employed	63 (19.2)	61 (18.9)		
Number of pre-pregnancy underlying diseases [*n* (%)]	None	250 (76.2)	233 (72.8)	2.607	0.456
	One	54 (16.5)	67 (20.9)		
	Two	20 (6.1)	20 (6.2)		
	Three or more	4 (1.2)	2 (0.6)		
Number of pregnancy complications [*n* (%)]	None	181 (55.1)	182 (56.9)	9.815	0.020
	One	90 (27.4)	108 (33.8)		
	Two	40 (12.2)	26 (8.1)		
	Three or more	17 (5.2)	6 (1.9)		
Number of anticipated reasons for discontinuing breastfeeding [*n* (%)]	One	255 (77.7)	265 (82.3)	4.403	0.221
	Two	50 (15.2)	44 (13.7)		
	Three or more	2 (0.6)	0 (0)		
	Never considered	21 (6.4)	13 (4.1)		
Number of channels for acquiring feeding knowledge [*n* (%)]	None	23 (7.0)	22 (6.9)	6.921	0.140
	One	155 (47.2)	142 (44.4)		
	Two	80 (24.4)	84 (26.2)		
	Three or more	70 (21.4)	74 (23.1)		
Primary postpartum caregiver [*n* (%)]	Postpartum care center	8 (2.4)	8 (2.5)	3.439	0.329
	Confinement nanny	54 (16.5)	63 (19.7)		
	Elderly family member	224 (68.3)	223 (69.7)		
	Husband or self	42 (12.8)	28 (8.8)		
Discussed feeding plan with spouse [*n* (%)]	No	83 (25.3)	100 (31.2)	2.657	0.103
	Yes	245 (74.7)	222 (68.8)		
Attention to infant formula information [*n* (%)]	No	166 (50.6)	153 (47.5)	0.622	0.430
	Yes	162 (49.4)	169 (52.5)		
Age (years)		29.5 (27, 32)	29 (27, 32)	−0.158	0.874
Social and professional support (SPS)		211 (153, 273)	123 (102, 176)	−12.052	<0.001
Positive breastfeeding attitude (PBS)		246 (208, 287)	230 (194, 265)	−4.021	<0.001
Negative breastfeeding attitude (NBS)		206 (172, 239)	208 (171, 238)	0.149	0.882
Behavioral control (BFC)		40 (35, 44)	36 (32, 40)	−5.765	<0.001
Total breastfeeding behavior score		16 (13, 18)	14 (12, 16)	−5.114	<0.001
Total infant feeding intention score		15 (12, 16)	12 (9, 15)	−7.803	<0.001
Total breastfeeding knowledge score		16 (14, 16.75)	15 (13, 16)	−4.138	<0.001
1-min Apgar score		10 (9, 10)	10 (10, 10)	1.491	0.136

### 3.2 Multivariate analysis

Multivariable logistic regression analysis was performed with the occurrence of non-exclusive breastfeeding within 6 weeks postpartum as the dependent variable. Variables with statistical significance in the univariable analyses (*p* < 0.05) were entered into the model as independent variables. Among these, SPS, PBS, BFC, total breastfeeding behavior score, total infant feeding intention score, and total breastfeeding knowledge score were entered as continuous variables using their original values. Immediate postpartum latching, history of breast surgery, amniotic fluid abnormality, and timely onset of lactogenesis II were entered as dichotomous variables. Number of pregnancy complications was treated as a multicategorical variable and entered into the logistic regression model using dummy variables.

The multivariable logistic regression analysis showed that immediate postpartum latching, amniotic fluid abnormality, SPS, PBS, total breastfeeding behavior score, total infant feeding intention score, and total breastfeeding knowledge score were independently associated with non-exclusive breastfeeding within 6 weeks postpartum (all *p* < 0.05). History of breast surgery showed borderline significance (*p* = 0.058), whereas timely onset of lactogenesis II and number of pregnancy complications were not statistically significant in the multivariable model (*p* > 0.05).

The final prediction model was established as follows: Logit(P) = 8.054–1.251 × (immediate postpartum latching) + 1.475 × (history of breast surgery) − 0.663 × (amniotic fluid abnormality) − 0.016 × (SPS score) + 0.005 × (PBS score) − 0.135 × (total breastfeeding behavior score) − 0.124 × (total infant feeding intention score) − 0.203 × (total breastfeeding knowledge score). The coding schemes for binary and dummy variables are presented in Table 2.

**Table 2:** Coding of independent variables included in the logistic regression model.

Independent variable	Coding method
Positive breastfeeding attitude (PBS)	Original value entered
Social and professional support (SPS)	Original value entered
Behavioral control (BFC)	Original value entered
Total breastfeeding behavior score	Original value entered
Total infant feeding intention score	Original value entered
Total breastfeeding knowledge score	Original value entered
Immediate postpartum latching	No = 0, Yes = 1
History of breast surgery	No = 0, Yes = 1
Amniotic fluid abnormality	No = 0, Yes = 1
Timely onset of lactogenesis ii	No = 0, Yes = 1
Number of pregnancy complications	Complication 1 (None = 0, One = 1, Two = 0, Three or More = 0)
	Complication 2 (None = 0, One = 0, Two = 1, Three or More = 0)
	Complication 3 (None = 0, One = 0, Two = 0, Three or More = 1)

The overall model fit was satisfactory. The likelihood ratio test showed that the model was statistically significant overall (χ^2^ = 258.368, *p* < 0.001), and the Hosmer–Lemeshow goodness-of-fit test yielded χ^2^ = 10.601 (*p* = 0.330), indicating good model fit. The coding of independent variables is shown in Table 2, and the results of the logistic regression analysis are presented in Table 3.

**Table 3:** Multivariable logistic regression analysis of factors associated with non-exclusive breastfeeding within 6 weeks postpartum among primiparous women.

Variable	Category	*B*	SE	Wald *χ*^*2*^	*P*	*OR*	95% CI	
							Lower limit	Upper limit
Immediate postpartum latching	No	1.251	0.282	19.700	< 0.001	3.492	2.010	6.067
	Yes							
History of breast surgery	No	−1.475	0.778	3.600	0.058	0.229	0.050	1.050
	Yes							
Amniotic fluid abnormality	No	0.663	0.249	7.062	0.008	1.940	1.190	3.162
	Yes							
Timely onset of lactogenesis II	No	0.112	0.205	0.297	0.586	1.118	0.748	1.670
	Yes							
Pregnancy complications (overall)				6.283	0.099			
Number of pregnancy complications (One vs. None)		0.422	0.547	0.595	0.441	1.525	0.522	4.460
Number of pregnancy complications (Two vs. None)		0.842	0.559	2.270	0.132	2.320	0.776	6.933
Number of pregnancy complications (Three or more vs. None)		0.135	0.624	0.047	0.829	1.144	0.337	3.885
Social and professional support (SPS)		−0.016	0.002	92.727	< 0.001	0.984	0.981	0.987
Positive breastfeeding attitude (PBS)		0.005	0.002	5.111	0.024	1.005	1.001	1.009
Total breastfeeding behavior score		−0.135	0.036	13.703	< 0.001	0.874	0.814	0.939
Total infant feeding intention Score		−0.124	0.031	15.844	< 0.001	0.883	0.831	0.939
Total breastfeeding knowledge score		−0.203	0.048	17.945	< 0.001	0.816	0.743	0.897
Constant		8.054	1.300	38.374	< 0.001	3146.280		

For dichotomous variables, the “Yes” category was treated as the reference category.

### 3.3 Predictive performance of the risk prediction model

**Figure 1:**
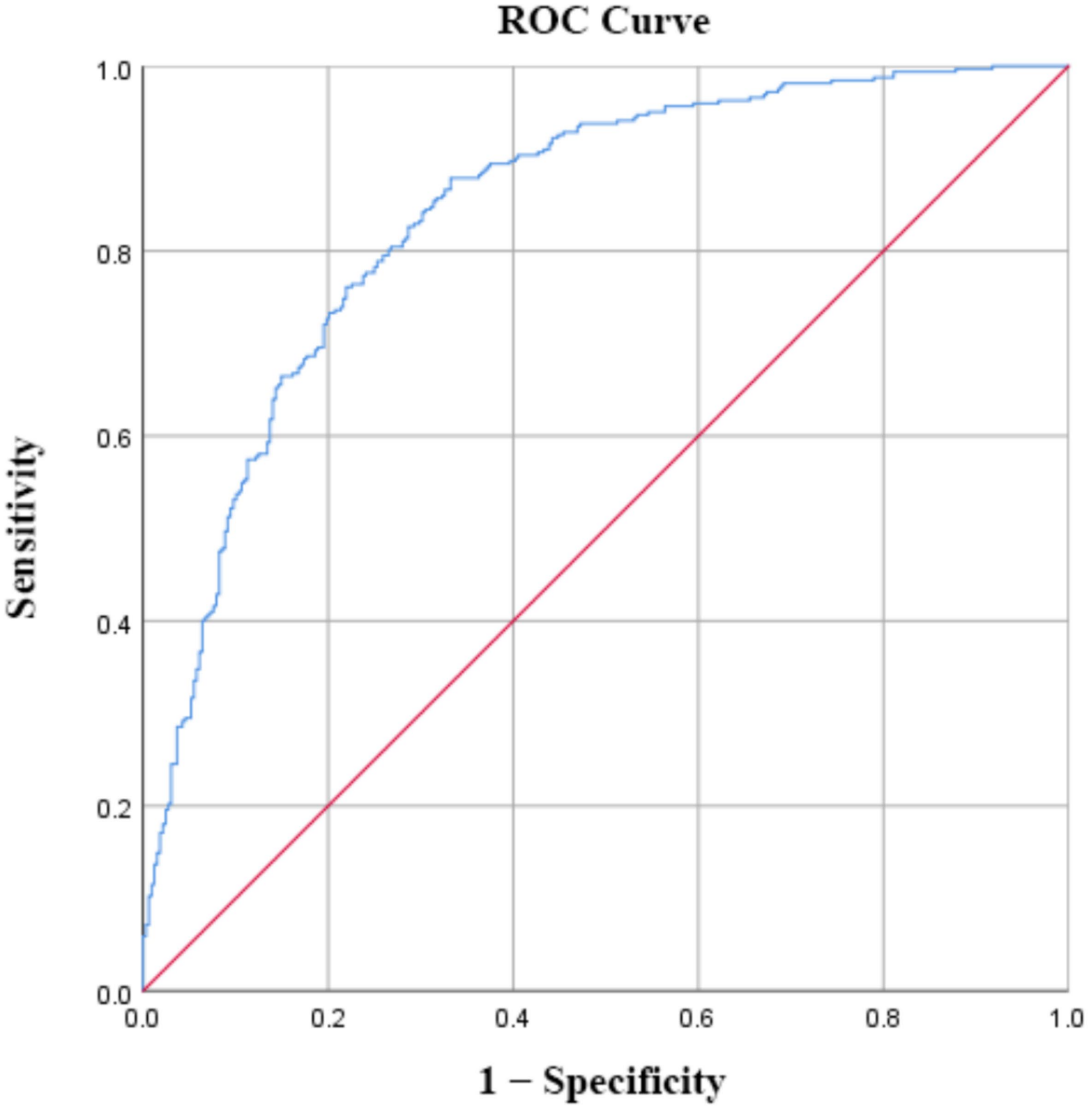
ROC curve of the logistic regression model.

The predictive performance of the model was evaluated using a ROC curve based on the predicted probabilities generated by the model, as shown in Figure 1. The logistic regression model yielded an AUC of 0.841 (95% confidence interval: 0.811–0.871), indicating good discriminative ability.

## 4 Discussion

In this cohort of 650 primiparous women, the incidence of non-exclusive breastfeeding within 6 weeks postpartum was 49.53%, indicating that nearly half of the mothers had already discontinued exclusive breastfeeding or introduced supplementary feeding during the early post-discharge period. Chen et al. reported that the exclusive breastfeeding rate at 6 weeks postpartum among Chinese women was 50.6%, indicating that the proportion of non-exclusive breastfeeding had already approached one half by this stage (21). Similarly, a prospective study by Rodríguez et al. in primiparous women showed a continuous decline in exclusive breastfeeding from hospitalization to 2 and 4 months postpartum, suggesting that breastfeeding failure is not mainly a late event but is concentrated in the first few weeks after birth (22). Taken together, these findings indicate that non-exclusive breastfeeding occurs predominantly in the early period after discharge, and that the first few postpartum weeks represent a critical window during which feeding patterns are most likely to change.

The final prediction model developed in this study incorporated clinically accessible variables reflecting early feeding initiation, amniotic fluid status, support resources, breastfeeding attitude, behavioral performance, feeding intention, and breastfeeding knowledge. Overall, the model showed satisfactory fit and good discriminative ability. Both the likelihood ratio test (*p* < 0.001) and the Hosmer–Lemeshow test (*p* = 0.330) supported adequate model fit, and the area under the receiver operating characteristic curve was 0.841, indicating that the model could effectively distinguish women at higher risk from those at lower risk. These findings suggest that early postpartum non-exclusive breastfeeding in primiparous women is not driven by any single demographic characteristic, but rather reflects the combined influence of perinatal experience, external support, breastfeeding attitude, knowledge, intention, and behavioral capacity. This is consistent with previous evidence that breastfeeding is a multifactorial behavioral outcome and underscores the need for risk identification based on integrated indicators rather than any single variable alone (23, 24).

Immediate postpartum latching was one of the most important perinatal factors identified in this study. Univariable analysis showed that the proportion of immediate postpartum latching was significantly higher in the exclusive breastfeeding group than in the non-exclusive breastfeeding group, suggesting that establishment of effective latching within the first hour after birth may directly influence the subsequent 6-week feeding trajectory. The World Health Organization recommends that breastfeeding should be initiated within 1 h after birth and emphasizes the importance of early, uninterrupted skin-to-skin contact and hands-on support in establishing breastfeeding (25). Keir et al. similarly reported that early breastfeeding initiation was closely associated with exclusive breastfeeding during hospitalization and with subsequent breastfeeding continuation (26). This may be because effective early latching promotes lactogenesis, improves mother–infant feeding coordination, and reduces the premature introduction of supplementary feeding due to perceived insufficient milk supply (27). In addition, successful first latching may itself serve as an important source of confidence for primiparous women (28). If effective latching is not established successfully in the immediate postpartum period, the negative effects of this early adverse feeding experience may persist even when subsequent education and support are provided (29).

A history of breast surgery showed borderline significance in relation to early postpartum non-exclusive breastfeeding in primiparous women, suggesting that prior breast-related surgical procedures may still be clinically relevant to the risk of non-exclusive breastfeeding. Compared with common variables such as immediate postpartum latching and feeding intention, a history of breast surgery has been reported relatively infrequently in predictive studies; however, its clinical significance should not be underestimated (30). A study by Mao et al. suggested that breastfeeding outcomes following breast surgery are closely related to the surgical technique and the extent of ductal and nerve preservation (31). Research by Faure et al. indicated that women who undergo breast reduction or related breast surgeries may experience a decrease in subsequent breastfeeding success rates and exclusive breastfeeding rates (32). This association may be related to factors such as damage to the mammary duct structure, altered nipple sensation, restricted milk ejection, and decreased maternal expectations of their own lactation ability. The findings of this study suggest that, in perinatal assessments, a history of breast surgery should not be simply regarded as general past medical history, but may still warrant attention as part of breastfeeding risk screening and individualized support planning.

Social and professional support was also associated with non-exclusive breastfeeding in primiparous women during the early postpartum period, indicating that breastfeeding outcomes are not solely a matter of personal choice, but also depend heavily on external support. The score for social and professional support was significantly higher in the exclusive breastfeeding group than in the non-exclusive breastfeeding group, suggesting that when support from family members, healthcare professionals, and peers is more adequate, mothers are more likely to maintain exclusive breastfeeding. This finding is consistent with previous research. In a randomized controlled trial involving primiparous women, Burns et al. found that telephone-based peer support significantly increased the rates of breastfeeding and exclusive breastfeeding continuation at 3 months postpartum (33). A systematic review and meta-analysis by Wong et al. focusing on primiparous women further indicated that educational support interventions spanning the antenatal to postpartum period, which are multi-component and combine face-to-face guidance with telephone follow-up, can increase exclusive breastfeeding rates at ≤2 months and 6 months (34). These findings suggest that the effectiveness of support interventions may not stem solely from one-time education, but rather depends on the continuous provision of problem identification, feeding technique guidance, emotional support, and decision-making support during the early postpartum period.

Positive breastfeeding attitude, breastfeeding behavior score, infant feeding intention score, and breastfeeding knowledge score were all associated with non-exclusive breastfeeding in the early postpartum period, indicating that feeding pattern transition is not attributable to a single factor, but results from the combined effects of attitude, knowledge, intention, and behavioral practice. Nommsen-Rivers et al. demonstrated in the development of the Infant Feeding Intentions scale that feeding intention intensity was significantly associated with subsequent exclusive breastfeeding duration (19). Oberfichtner et al. noted that the influence of breastfeeding knowledge on feeding duration was particularly pronounced among primiparous women, whereas this association was less prominent among multiparous women (35). Manjapallikkunnel et al. also demonstrated that higher breastfeeding knowledge levels and positive feeding attitudes were closely associated with the maintenance of exclusive breastfeeding postpartum (36). For primiparous women, breastfeeding knowledge, attitude, intention, and behavioral capacity represent the cognitive foundation, psychological readiness, and practical execution required for maintaining breastfeeding (37). A weakness in any of these domains may reduce the likelihood of maintaining exclusive breastfeeding when feeding difficulties arise in the early postpartum period. Therefore, the significance of the present study lies not only in identifying factors associated with non-exclusive breastfeeding in the early postpartum period, but also in suggesting that its occurrence is often the result of the combined effects of multiple interrelated factors, including attitude, cognition, intention, and behavioral practice.

From a practical perspective, most variables included in the model established in this study can be obtained during hospitalization or prior to discharge, which enables early identification and proactive intervention. In other words, the core value of this model does not lie merely in achieving a satisfactory area under the curve statistically, but in helping clinicians identify high-risk individuals before actual breastfeeding failure occurs and prioritize limited breastfeeding support resources for those in greatest need. Based on the results of this study, the key management strategies for high-risk primiparous women should include strengthening effective latching support within 1 h after birth during the perinatal period, improving maternal breastfeeding knowledge, positive breastfeeding attitudes, behavioral skills, and feeding intention during hospitalization, and establishing a continuous family–hospital support pathway during the first 6 weeks after discharge. Additional attention may also be warranted for women with abnormal perinatal clinical conditions, including amniotic fluid abnormalities. This intervention orientation is consistent with findings from previous studies on supportive interventions for primiparous women, indicating that interventions should cover the prenatal to postpartum period and emphasize multi-component integration and continuous follow-up (34, 38).

## 5 Conclusion

This study indicates that the incidence of non-exclusive breastfeeding among primiparous women within 6 weeks postpartum is relatively high, and that the early postpartum period represents a critical stage during which exclusive breastfeeding is most likely to be discontinued. Immediate postpartum latching, amniotic fluid abnormality, social and professional support, positive breastfeeding attitude, breastfeeding-related behavioral performance, infant feeding intention, and breastfeeding knowledge were important factors associated with non-exclusive breastfeeding within 6 weeks postpartum. The risk prediction model constructed on the basis of these variables showed good fit and satisfactory discriminative ability, and may provide a basis for early risk stratification of non-exclusive breastfeeding among primiparous women, as well as a reference for clinical implementation of early screening, stratified follow-up, and targeted support.

## Data Availability

The raw data supporting the conclusions of this article will be made available by the authors, without undue reservation.
